# ﻿Taxonomic notes on four closely related spider species of the genus *Khorata* (Araneae, Pholcidae)

**DOI:** 10.3897/zookeys.1244.154422

**Published:** 2025-07-11

**Authors:** Jinglin Li, Baisensen Zhang, Shuqiang Li, Zhiyuan Yao

**Affiliations:** 1 College of Life Science, Shenyang Normal University, Shenyang 110034, China Shenyang Normal University Shenyang China; 2 Institute of Zoology, Chinese Academy of Sciences, Beijing 100101, China Institute of Zoology, Chinese Academy of Sciences Beijing China

**Keywords:** Asia, Asian cellar spiders, biodiversity, distribution, key, morphology, new species, Pholcinae, species grouping, taxonomy

## Abstract

*Khorata* Huber, 2005 contains 53 species. It is distributed in the Indo-Malayan region. In this study, a new species is described from Guangxi, China: *Khorataqianlei* Li, Li & Yao, **sp. nov.** (♂♀). This new species is similar to *K.digitata* Yao & Li, 2010 (Guangxi, China; Vietnam), *K.qian* Yao & Li, 2019 (Guizhou, China), and *K.yuhaoi* Xu, Zheng & Yao, 2020 (Guizhou and Yunnan, China). Taxonomic keys for distinguishing these four closely related species and a distribution map of all species in this genus are provided. In addition, 37 species of this genus are classified into nine formal species groups.

## ﻿Introduction

The genus *Khorata* Huber, 2005 (Pholcinae C.L. Koch, 1850) includes 53 valid species, with the highest diversity in southern China (35 spp.), followed by Vietnam (10), Laos (5), Thailand (4), and Cambodia (1) (e.g., Huber 2005; Zhang and Zhang 2008; Zhang and Zhu 2009; Yao and Li 2010, 2013; Yao et al. 2014, 2015, 2019; Nie et al. 2018; Xu et al. 2020; Lan et al. 2021; WSC 2025). This genus has not yet been divided into species groups, and no study has focused on its phylogeny.

Of the 53 *Khorata* species, the greatest diversity occurs in Guangxi, China, with 20 species documented in this region. This study describes a *Khorata* species from Guangxi, collected from interstitial rock habitats. To facilitate identification, we provide taxonomic keys distinguishing this new species from three morphologically similar congeners. A comprehensive distribution map is included, illustrating the geographic ranges of all 54 *Khorata* species. Additionally, nine species groups are proposed for 37 species.

## ﻿Material and methods

Specimens were examined and measured with a Leica M205 C stereomicroscope. The left male palp was photographed. The epigyne was photographed before dissection. The endogyne was photographed after being treated in a 10% warm solution of potassium hydroxide (KOH) to dissolve soft tissues. Images were captured with a Canon EOS 750D wide zoom digital camera (24.2 megapixels) mounted on the stereomicroscope mentioned above and assembled using Helicon Focus v. 3.10.3 image-stacking software (Khmelik et al. 2005). All measurements are given in millimeters (mm). Leg measurements are shown as: total length (femur, patella, tibia, metatarsus, tarsus). Leg segments were measured on their dorsal sides. The distribution map was generated with ArcGIS v. 10.2 (ESRI Inc.). The specimens studied are preserved in 75% ethanol and deposited in the College of Life Science, Shenyang Normal University (SYNU) in Liaoning, China.

Terminology and taxonomic descriptions follow Huber (2005), Yao et al. (2015), and Xu et al. (2020). The following abbreviations are used: **ALE** = anterior lateral eye, **L/d** = length/diameter, **PME** = posterior median eye.

## ﻿Taxonomy

### ﻿Family Pholcidae C.L. Koch, 1850

**Subfamily Pholcinae C.L. Koch, 1850**

#### ﻿Genus Khorata

Taxon classificationAnimaliaAraneaePholcidae

Huber, 2005

88ED1591-BFA4-5D14-B976-0A840B58C4F1

Khorata Huber, 2005: 79. Yao and Li 2010: 5. Xu et al. 2020: 159.

##### Type species.

*Khoratakhammouan* Huber, 2005 from Laos.

##### Diagnosis.

The genus can be distinguished from *Savarna* Huber, 2005 by the male chelicera with a sclerotized distal apophysis (da) on the front-lateral surface and a hooked frontal apophysis (fa2), by the male palpal trochanter apophysis extremely short (as long as wide) and not attached to the femur, by the male palpal femur with a retrolateral apophysis (ra), and by the epigyne with a posterior lip (pl) in nearly half of the species.

##### Remarks.

**Figure 1.**
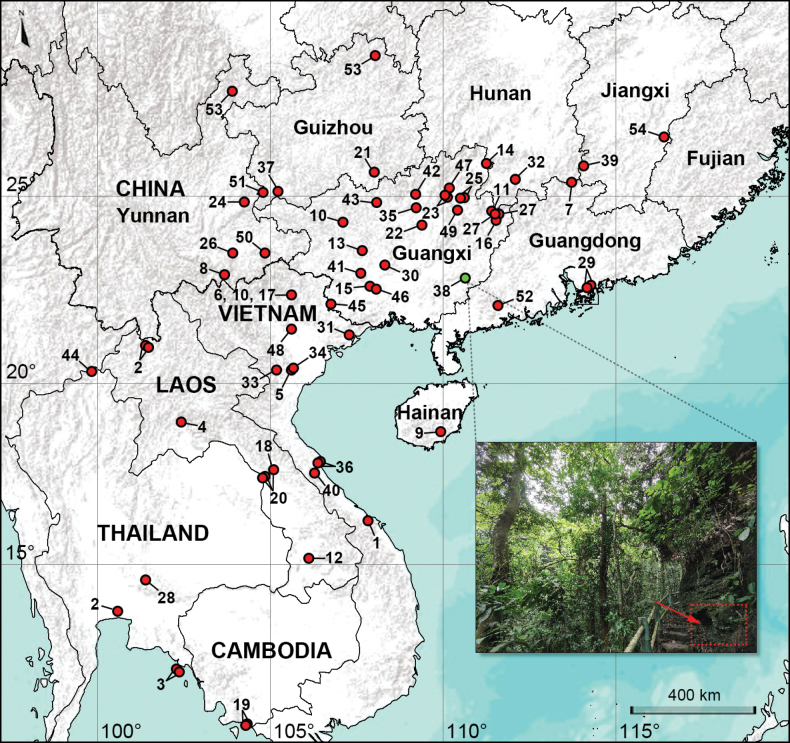
Distribution records of *Khorata* species. **1***K.bachma* Yao & Li, 2018 **2***K.bangkok* Huber, 2005 **3***K.bayeri* Yao, Li & Jäger, 2014 **4***K.circularis* Yao & Li, 2013 **5***K.cucphuong* Yao & Li, 2018 **6***K.dangi* Yao, Pham & Li, 2015 **7***K.danxia* Sheng & Xu, 2021 **8***K.dawei* Yao & Li, 2019 **9***K.diaoluoshanensis* Tong & Li, 2008 **10***K.digitata* Yao & Li, 2010 **11***K.dongkou* Yao & Li, 2010 **12***K.dupla* Yao & Li, 2013 **13***K.epunctata* Yao & Li, 2010 **14***K.flabelliformis* Yao & Li, 2010 **15***K.fusui* Zhang & Zhu, 2009 **16***K.guiensis* Yao & Li, 2010 **17***K.huberi* Yao, Pham & Li, 2015 **18***K.jaegeri* Huber, 2005 **19***K.kep* Lan, Jäger & Li, 2021 **20***K.khammouan* Huber, 2005 **21***K.libo* Yao & Li, 2019 **22***K.liuzhouensis* Yao & Li, 2010 **23***K.luojinensis* Yao & Li, 2010 **24***K.luoping* Yao & Li, 2019 **25***K.macilenta* Yao & Li, 2010 **26***K.matang* Yao & Li, 2019 **27***K.miaoshanensis* Yao & Li, 2010 **28***K.musee* Lan & Li, 2021 **29***K.nani* Xu, Zheng & Yao, 2020 **30***K.nanningensis* Yao & Li, 2010 **31***K.ningming* Zhang & Zhu, 2009 **32***K.ningyuan* Wei & Xu, 2014 **33***K.ninhbinh* Zhang, Li & Yao, 2024 **34***K.palace* Yao & Li, 2018 **35***K.paquini* Yao & Li, 2010 **36***K.protumida* Yao, Pham & Li, 2015 **37***K.qian* Yao & Li, 2019 **38***K.qianlei* sp. nov. **39***K.qiyunshanensis* Zhou, 2024 **40***K.quangbinh* Yao & Li, 2018 **41***K.robertmurphyi* Yao & Li, 2010 **42***K.rongshuiensis* Yao & Li, 2010 **43***K.sancai* Wei & Xu, 2014 **44***K.schwendingeri* Huber, 2005 **45***K.shao* Yao & Li, 2010 **46***K.suwei* Yao & Li, 2019 **47***K.triangula* Yao & Li, 2010 **48***K.vinhphuc* Yao & Li, 2018 **49***K.wangae* Yao & Li, 2010 **50***K.wenshan* Yao & Li, 2019 **51***K.xingyi* Chen, Zhang & Zhu, 2009 **52***K.yangchun* Yao & Li, 2019 **53***K.yuhaoi* Xu, Zheng & Yao, 2020 **54***K.zhui* Zhang & Zhang, 2008. Arrow indicates the habitat.

Species of *Khorata* are medium-sized with long legs, and construct domed webs at limestone cave entrances or between rocks (Xu et al. 2020). Three notable exceptions, *K.dupla* Yao & Li, 2013, *K.bayeri* Yao, Li & Jäger, 2014 and *K.kep* Lan, Jäger & Li, 2021, possess shorter legs and inhabit leaf litter (Yao and Li 2013; Yao et al. 2014; Lan et al. 2021). At present, the records from Kep (Cambodia; *K.kep*), Chiang Rai (Thailand; *K.schwendingeri* Huber, 2005), Tongren (Guizhou, China; *K.yuhaoi* Xu, Zheng & Yao, 2020) and Wuyi Mt. (Fujian, China; *K.zhui* Zhang & Zhang, 2008) represent the southernmost, westernmost, northernmost and easternmost distribution limit for the genus, respectively (Fig. 1). Forty-two species (79%) are known from a single locality and three species are sympatric (found in the same locality; *K.dangi* Yao, Pham & Li, 2015, *K.digitata* Yao & Li, 2010, *K.huberi* Yao, Pham & Li, 2015). Morphological data indicate a close relationship among the genera *Khorata*, *Savarna*, *Hantu* Huber, 2016, and *Aetana* Huber, 2005 (Huber et al. 2015); however, the first three genera were merely used as outgroups for the genus *Aetana*, and the evidence supporting the inter-genetic relationships was limited. The most recent molecular data suggest that the phylogenetic positions of *Khorata*, *Savarna*, and *Hantu* within the ‘Pholcinae group 1’ are unstable and problematic, necessitating further analysis (Eberle et al. 2018; Huber et al. 2018).

Thirty-seven species of this genus are here divided into nine formal species groups based on superficial similarity, while the remaining species are tentatively not assigned to any species groups.

**Figure 2.**
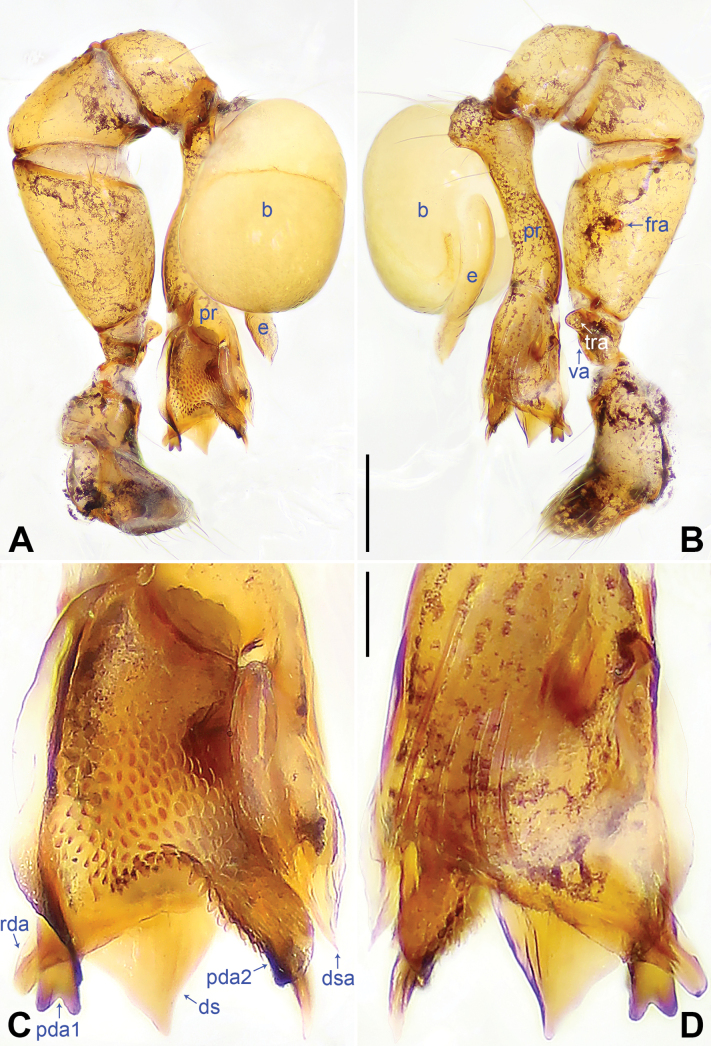
*Khorataqianlei* sp. nov., holotype male. **A, B.** Palp (**A.** Prolateral view; **B.** Retrolateral view); **C, D.** Distal part of procursus (**C.** Prolateral view; **D.** Retrolateral view). Abbreviations: b = bulb, ds = distal sclerite, dsa = dorso-subdistal apophysis, e = embolus, fra = femoral retrolateral apophysis, pda = prolatero-distal apophysis, pr = procursus, rda = retrolatero-distal apophysis, tra = trochanteral retrolateral apophysis, va = ventral apophysis. Scale bars: 0.20 mm (**A, B**); 0.05 mm (**C, D**).

1. The *digitata* group. This group includes four species: *K.digitata*, *K.qian* Yao & Li, 2019, *K.qianlei* sp. nov. and *K.yuhaoi*. They share five putative synapomorphies on the procursus: two prolatero-distal apophyses (pda1, pda2), distal sclerite (ds), dorso-subdistal apophysis (dsa), and retrolatero-distal apophysis (rda) (Figs 2C, 4A, C, E). In addition, endogynes of *K.digitata*, *K.qian* and *K.yuhaoi* have sclerites (as) anterior to pore plates (pp) (Fig. 4B, D, F).
2. The *epunctata* group. This group includes four species: *K.circularis* Yao & Li, 2013, *K.epunctata* Yao & Li, 2010, *K.fusui* Zhang & Zhu, 2009 and *K.shao* Yao & Li, 2010. They share two putative synapomorphies on the procursus: lamellar ventro-distal apophysis (vda) and slightly sclerotized distal apophysis (da) (e.g., fig. 28C in Yao and Li 2013).
3. The *flabelliformis* group. This group includes six species: *K.dongkou* Yao & Li, 2010, *K.flabelliformis* Yao & Li, 2010, *K.guiensis* Yao & Li, 2010, *K.macilenta* Yao & Li, 2010, *K.miaoshanensis* Yao & Li, 2010 and *K.ningyuan* Wei & Xu, 2014. They share two putative synapomorphies: spine-shaped prolatero-ventral apophysis (pva) on the procursus (e.g., fig. 13D in Yao and Li 2010) and posterior lip (pl) on the epigyne (e.g., fig. 14A in Yao and Li 2010).
4. The *khammouan* group. This group includes two species: *K.khammouan* Huber, 2005 and *K.protumida* Yao, Pham & Li, 2015. They share two putative synapomorphies: sclerotized prolatero-dorsal apophysis (pda) on the procursus (e.g., fig. 151 in Huber 2005) and a pair of protrusions on the epigyne (e.g., fig. 155 in Huber 2005).
5. The *matang* group. This group includes two species: *K.matang* Yao & Li, 2019 and *K.wenshan* Yao & Li, 2019. They share three putative synapomorphies: bifurcated prolatero-distal apophysis (pda) and bifurcated retrolatero-distal apophysis (rda) on the procursus (e.g., fig. 7C, D in Yao et al. 2019) and posterior lip (pl) on the epigyne (e.g., fig. 8A in Yao et al. 2019).
6. The *nanningensis* group. This group includes eight species: *K.bachma* Yao & Li, 2018, *K.danxia* Sheng & Xu, 2021, *K.kep*, *K.nanningensis* Yao & Li, 2010, *K.ninhbinh* Zhang, Li & Yao, 2024, *K.quangbinh* Yao & Li, 2018, *K.robertmurphyi* Yao & Li, 2010 and *K.suwei* Yao & Li, 2019. They share two putative synapomorphies on the procursus: lamellar ventro-distal apophysis (vda) and dorso-distal apophysis (dda) bearing teeth (e.g., fig. 37D in Yao and Li 2010).
7. The *palace* group. This group includes two species: *K.luoping* Yao & Li, 2019 and *K.palace* Yao & Li, 2018. They share three putative synapomorphies on the procursus: bifurcated prolatero-distal apophysis (pda), ventro-distal apophysis (vda), and spine-shaped retrolatero-distal apophysis (rda) (e.g., fig. 7C in Nie et al. 2018).
8. The *schwendingeri* group. This group includes three species: *K.bangkok* Huber, 2005, *K.musee* Lan & Li, 2021 and *K.schwendingeri*. They share one putative synapomorphy on the procursus: spine-shaped prolatero-dorsal apophysis (pda) (e.g., fig. 161 in Huber 2005).
9. The *triangula* group. This group includes six species: *K.libo* Yao & Li, 2019, *K.liuzhouensis* Yao & Li, 2010, *K.luojinensis* Yao & Li, 2010, *K.paquini* Yao & Li, 2010, *K.sancai* Wei & Xu, 2014 and *K.triangula* Yao & Li, 2010. They share one putative synapomorphy on the procursus: lamellar distal apophysis (da) (e.g., fig. 55D in Yao and Li 2010).

### ﻿Identification keys to the *digitata* group

#### ﻿Males

**Table:** 

1	Procursus (pr) with bifurcated prolatero-distal apophysis (pda1) (Fig. 2C); male cheliceral frontal apophyses (fa1) wedge-shaped (pointed in lateral view, Fig. 3D)	***K.qianlei* sp. nov.**
–	Procursus (pr) with blunt prolatero-subdistal apophysis (psa) (Fig. 4A, C, E); male cheliceral frontal apophyses blunt (fig. 3B in Yao and Li 2010, arrow in fig. 10D in Yao et al. 2019, arrow in fig. 6D in Xu et al. 2020)	**2**
2	Angular distal sclerite (ds) of procursus longer than wide (Fig. 4A); pointed dorso-subdistal apophysis (dsa) of procursus shorter than blunt prolatero-distal apophysis (pda) (Fig. 4A)	***K.digitata***
–	Angular distal sclerite (ds) of procursus as wide as long (Fig. 4C, E); pointed dorso-subdistal apophysis (dsa) of procursus as long as blunt prolatero-distal apophysis (pda) (Fig. 4C, E)	**3**
3	Blunt retrolatero-distal apophysis (rda) of procursus longer than angular distal sclerite (ds) (Fig. 4C)	***K.qian***
–	Blunt retrolatero-distal apophysis (rda) of procursus as long as angular distal sclerite (ds) (Fig. 4E)	***K.yuhaoi***

#### ﻿Females

**Table:** 

1	Epigynal plate posteriorly slightly curved (arrow in Figs 3B, 4F)	**2**
–	Epigynal plate with posterior lip (pl) (Fig. 4B, D)	**3**
2	Pore plates (pp) nearly triangular (Fig. 3B); anterior arch (aa) without sclerites (Fig. 3B)	***K.qianlei* sp. nov.**
–	Pore plates (pp) nearly elliptical (Fig. 4F); anterior arch (aa) with sclerites (as) anterior to pore plates (pp) (Fig. 4F)	***K.yuhaoi***
3	Pore plates (pp) nearly triangular (Fig. 4B); sclerites of anterior arch (as) as long as wide (Fig. 4B)	***K.digitata***
–	Pore plates (pp) nearly elliptical (Fig. 4D); sclerites of anterior arch (as) wider than long (Fig. 4D)	***K.qian***

##### ﻿ Khorata qianlei

Taxon classificationAnimaliaAraneaePholcidae

Li, Li & Yao sp. nov.

DF88E5AB-C2E1-5000-AD51-92397912BF9B

https://zoobank.org/2A5EDE44-0138-4839-9112-DDEE9ACA0EFD

Figs 2 , 3

###### Type material.

***Holotype*: China** • ♂; Guangxi Prov., Yulin, Rong Co., Duqiaoshan Forest Park; 22.821559°N, 110.642871°E; alt. 187 m; 5 Oct. 2023; Q. Lu leg.; SYNU-Ar00493. ***Paratypes*: China** • 1♂; same data as for the holotype; SYNU-Ar00494 • 1♀; same data as for the holotype; SYNU-Ar00495.

###### Etymology.

The specific name is a patronym in honor of the collector Qianle Lu (Shenzhen, China).

###### Diagnosis.

**Figure 3.**
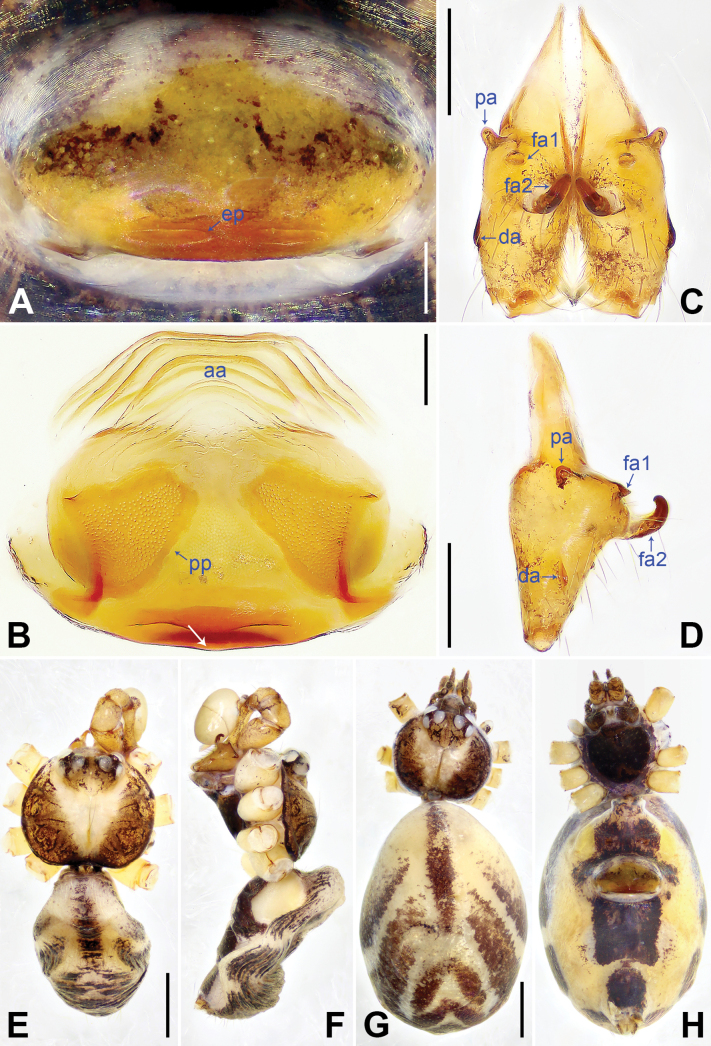
*Khorataqianlei* sp. nov., holotype male (**C–F**) and paratype female (**A, B, G, H**). **A.** Epigyne; **B.** Endogyne, arrow points at slightly curved part; **C, D.** Chelicerae (**C.** Frontal view; **D.** Lateral view); **E–H.** Habitus (**E, G.** Dorsal view; **F.** Lateral view; **H.** Ventral view). Abbreviations: aa = anterior arch, da = distal apophysis, ep = epigynal pocket, fa = frontal apophysis, pa = proximo-lateral apophysis, pp = pore plate. Scale bars: 0.10 mm (**A, B**); 0.20 mm (**C, D**); 0.50 mm (**E–H**).

**Figure 4.**
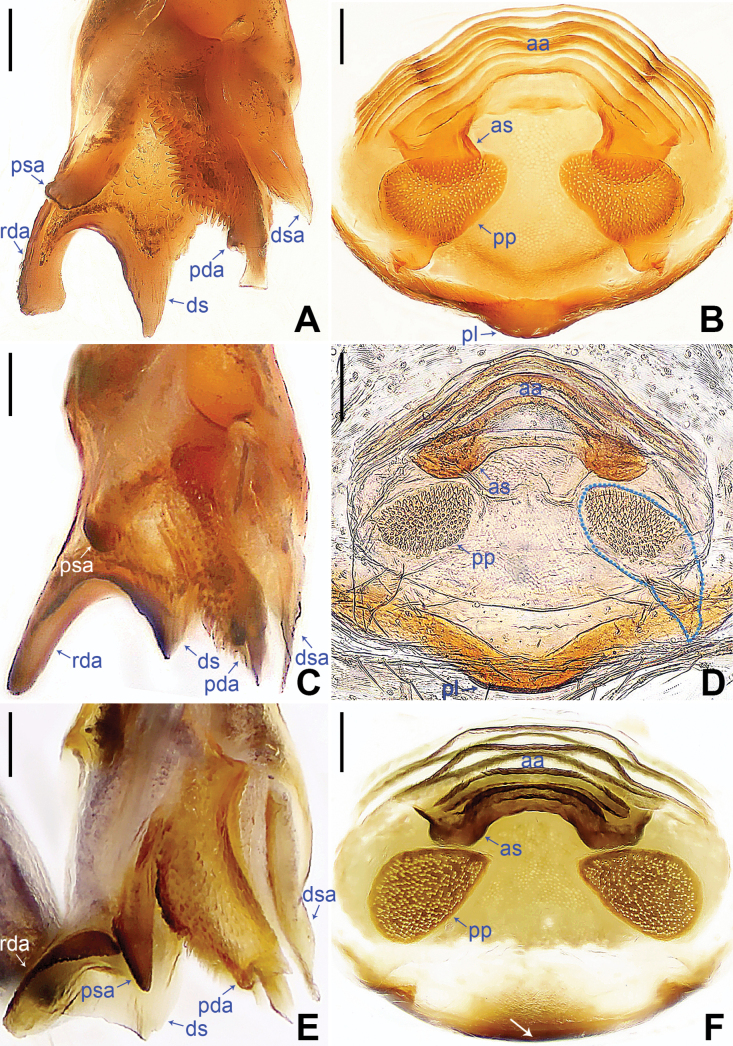
*Khoratadigitata* (**A, B**), *K.qian* (**C, D**), *K.yuhaoi* (**E, F**), male (**A, C, E**) and female (**B, D, F**). **A, C, E.** Distal parts of procursus, prolateral views; **B, D, F.** Endogynes (**F.** Arrow points at slightly curved part). Abbreviations: aa = anterior arch, as = anterior sclerite, ds = distal sclerite, dsa = dorso-subdistal apophysis, pda = prolatero-distal apophysis, pl = posterior lip, pp = pore plate, psa = prolatero-subdistal apophysis, rda = retrolatero-distal apophysis. Scale bars: 0.05 mm (**A, C, E**); 0.10 mm (**B, D, F**).

The new species can be distinguished from *K.yuhaoi* by the procursus with a bifurcated prolatero-distal apophysis (pda1) (Fig. 2C vs psa not bifurcated, Fig. 4E), by the male cheliceral frontal apophyses (fa1) wedge-shaped (pointed in lateral view, Fig. 3D vs blunt, arrow in fig. 6D in Xu et al. 2020), by the anterior arch (aa) lacking sclerites (as) (Fig. 3B vs present, Fig. 4F), and by the pore plates (pp) nearly triangular (Fig. 3B vs nearly elliptical, Fig. 4F).

###### Description.

**Male** (***holotype***): Measurements: Total length 2.28 (2.34 with clypeus), carapace 0.87 long, 1.04 wide, opisthosoma 1.41 long, 0.91 wide. Leg I: 22.69 (5.51, 0.43, 5.45, 8.72, 2.58), leg II: 14.23 (4.00, 0.41, 3.28, 4.94, 1.60), leg III: 10.60 (3.08, 0.38, 2.38, 3.76, 1.00), leg IV: 13.82 (4.15, 0.39, 3.16, 5.10, 1.02); tibia I L/d: 61. Eye sizes and interdistances: PME–PME 0.10, PME 0.13, PME–ALE 0.04. Sternum width/length: 0.65/0.52.

***Color***: Carapace yellowish, with dark brown lateral margins and narrow, dark median line; sternum brown. Legs brownish, slightly whitish on distal parts of femora and tibiae, with darker rings on subdistal parts of femora and proximal and subdistal parts of tibiae. Opisthosoma yellowish, with dark brown bands.

***Body*** (Fig. 3E, F): Thoracic furrow shallow, but distinct; clypeus unmodified.

***Chelicera*** (Fig. 3C, D) with 4 apophyses: Proximo-lateral (pa), distal on front-lateral surface (da), wedge-shaped frontal (fa1), and hooked frontal (fa2).

***Palp*** (Fig. 2A–D): Trochanter with short retrolateral apophysis (tra) (as long as wide) and ventral apophysis (va); femur 1.5× longer than patella and 2× longer than tibia; femur with retrolateral apophysis (fra); procursus (pr) simple proximally but complex distally, with bifurcated prolatero-distal apophysis (pda1), angular distal sclerite (ds), blunt prolatero-distal apophysis (pda2) (bearing pointed branch), pointed dorso-subdistal apophysis (dsa), and blunt retrolatero-distal apophysis (rda); bulb simple, no other apophyses except for embolus; embolus distally pointed, 8× longer than wide.

***Legs***: Retrolateral trichobothrium on tibia I situated at 4% proximally; legs with short erected setae on metatarsi and tarsi; tarsus I with 16 distinct pseudosegments.

**Female** (***paratype***, SYNU-Ar00495): Similar to male, habitus as in Fig. 3G, H. Total length 3.13 (3.26 with clypeus), carapace 0.80 long, 0.92 wide, opisthosoma 2.33 long, 1.50 wide; tibia I: 4.40; tibia I L/d: 55. Eye sizes and interdistances: PME–PME 0.08, PME 0.12, PME–ALE 0.03. Sternum width/length: 0.65/0.63. Epigyne (Fig. 3A) externally elliptical (2× wider than long), posteriorly slightly curved, with pair of posterior pockets (ep) 0.05 apart. Endogyne (Fig. 3B) with curved anterior arch (aa) and pair of nearly triangular pore plates (pp) on lateral part.

###### Variation.

Tibia I in male paratype (SYNU-Ar00494): 5.38.

###### Habitat.

The species was found on the web between rocks in primary forest.

###### Distribution.

China (Guangxi, type locality; Fig. 1).

## Supplementary Material

XML Treatment for Khorata

XML Treatment for Khorata qianlei

## ﻿Additional information

### Use of AI

No use of AI was reported.

### Author ORCIDs

Jinglin Li https://orcid.org/0009-0008-3932-309X

Shuqiang Li https://orcid.org/0000-0002-3290-5416

Zhiyuan Yao https://orcid.org/0000-0002-1631-0949

### Data availability

All of the data that support the findings of this study are available in the main text.
